# Smoking among adolescents in Northern Greece: a large cross-sectional study about risk and preventive factors

**DOI:** 10.1186/1747-597X-7-38

**Published:** 2012-09-10

**Authors:** Dionisios G Spyratos, Despoina T Pelagidou, Diamantis Chloros, Anna-Bettina Haidich, Eleni Karetsi, Christina Koubaniou, Stavros Konstantopoulos, Konstantinos Gourgoulianis, Lazaros T Sichletidis

**Affiliations:** 1 Pulmonary Department, Aristotle University of Thessaloniki, Thessaloniki, Greece; 2 Pulmonary Department, University of Thessaly, Larisa, Greece; 3 Pulmonary Department, University of Ioannina, Ioannina, Greece

## Abstract

**Background:**

The aim of the present study was to investigate epidemiological data about cigarette smoking in relation with risk and preventive factors among Greek adolescents.

**Methods:**

We randomly selected 10% of the whole number of schools in Northern Greece (133 schools, 18,904 participants were included). Two anonymous questionnaires (smoker's and non-smoker's) were both distributed to all students so they selected and filled in only one. A parental signed informed consent was obtained using an informative leaflet about adolescent smoking.

**Results:**

The main findings of the study were: a) 14.2% of the adolescents (mean age+/−SD: 15.3+/−1.7 years) reported regular smoking (24.1% in the age group 16–18 years), b) 84.2% of the current smokers reported daily use, c) students who live in urban and semirural areas smoke more frequently than those in rural areas, d) students in technically oriented schools smoke twice as frequent compared to those in general education, e) risk factors for smoking: male gender, low educational level of parents, friends who smoke (OR: 10.01, 95%CI: 8.53-11.74, p<0.001), frequent visits to internet cafes (OR:1.53, 95%CI: 1.35-1.74, p<0.001), parents, siblings (OR:2.24, 95%CI: 1.99-2.51, p<0.001) and favorite artist (OR:1.18, 95%CI: 1.04-1.33, p=0.009) who smoke, f) protective factors against smoking: participation in sports (OR:0.59, 95%CI: 0.53-0.67, p<0.001), watching television (OR:0.74, 95%CI 0.66-0.84, p<0.001) and influence by health warning messages on cigarette packets (OR:0.42, 95%CI: 0.37, 0.48, p<0.001).

**Conclusions:**

Even though prevalence of cigarette smoking is not too high among Greek adolescents, frequency of everyday cigarette use is alarming. We identified many social and lifestyle risk and preventive factors that should be incorporated in a national smoking prevention program among Greek adolescents.

## Background

Cigarette smoking is considered a modern epidemic with incalculable consequences for public health and economy. It was estimated that in 2008, smoking was responsible for the death of more than 5 million people worldwide - more than those caused by tuberculosis, AIDS and malaria together - and it is expected that in the 21^st^ century this number will approach 1 billion unless antismoking measures will be taken [1]. The use of tobacco is the world's leading cause of morbidity and premature mortality that can be prevented [2].

Smoking experimentation is related to adolescence as adolescents are more influenced by their social environment [3]. Almost all adults who smoke regularly report that they started at or before age 18 [4], adolescents are less likely to quit smoking [5,6] as symptoms of nicotine dependence can easily be developed [7]. The prevalence of current smoking among adolescents in the USA and in many European countries is well above the established targets for the near future [8,9].

Many social and psychological factors have been positively related with adolescent smoking: parental and peer smoking [10,11], poor performance in school [12], low socioeconomic conditions [13,14], childhood abuse [15,16], exposure to films or advertisements which promote smoking [17-20], anxiety [21] and depression [22]. On the other hand there are some protective factors even though data are not so robust. Parents or friends who do not smoke [23,24], negative beliefs about smoking [24], sports participation [25] and religious identity [26].

The aim of the present study was to investigate epidemiological trends about cigarette smoking as well as psychosocial differences between adolescents (12–18 years) smokers and non-smokers that may act as preventive or risk factors. A secondary aim of the study was the validation of two questionnaires – one for smokers and another one for non-smokers – in a subgroup of the initial sample.

## Methods

### Design

Initially, a special proposal-application was submitted to the Ministry of Education, requesting permission so that a questionnaire on smoking habits may be answered by students (12–18 year old) in junior high and high schools of Northern Greece. After the approval was obtained, we used summary lists of all junior high and high schools in the prefectures of Macedonia (13 counties), Thrace (3 counties), Thessaly (4 counties) and Epirus (4 counties). The exact number of schools, the type of school (e.g. general or technical education, morning or evening shift) and the number of students was reported.

This is a surveillance study with a reference population consisted of 1,341 schools. Then we excluded schools for children with mental disabilities, church-schools and evening schools that enrolled mostly adults. We randomly selected 10% of the original sample of schools and the present study was conducted in 133 schools. The degree of reproducibility was assessed by recompletion of the questionnaires – over a period of two weeks – in 10% of the schools that had been selected (13 schools). Finally we distributed the questionnaires to the whole sample of the study which was conducted from November 2009 to May 2010.

### Questionnaires

A parental signed informed consent was obtained using an informative leaflet about adolescent smoking and the questionnaire their children are going to complete. For the purpose of the study two questionnaires were prepared: one for smokers and the other one for non-smokers (see Additional file 1). The questionnaires were anonymous, were both distributed simultaneously to all students so they selected and filled in only one of them.

The students completed the questionnaires in the classroom under examination conditions, the teacher was absent while the researcher explained in detail all questions and the children wrote their answers. At the end of the survey, each child sealed the completed questionnaire in an envelope. This whole procedure was designed in such a way to emphasize that their answers were confidential. The main advantage of the questionnaires was that the average time to be completed was 10–15 minutes. After completion of the questionnaires, doctors who took part in the study gave lectures about the consequences of cigarette smoking on human health and the importance of early cessation.

Questions about anthropometric data, patterns of smoking behaviour, parents’ educational level, smoking habits of family and social environment, leisure activities, possible causes of initiation or abstinence, proposals for anti-smoking measures were included. The majority of the questions had predefined answers to promote convenience and participation while several questions were the same in both questionnaires. Current cigarette smoker was considered every participant who had smoked at least one cigarette during the month before completing the questionnaire.

### Statistical analysis

Reproducibility for each question was assessed using the Cohen’s kappa statistic. As an overall rate of reproducibility for the entire questionnaire the mean of the Cohen’s kappa statistic and the corresponding 95% confidence intervals were reported. Initially, the test Kolmogorov-Smirnov was used in the quantitative variables to determine if the data was normally distributed. The percentages were compared by chi-square test. Student’s *t* test was applied to compare two groups when data were normally distributed; otherwise the corresponding non-parametric Mann–Whitney *U* test was applied. The analysis of variance (ANOVA) or the corresponding non-parametric Kruskal-Wallis test was used to compare groups of three or more. The association of smoking with other factors was estimated with random-effects logistic regression in order to take into account the clustering of schools. First univariate analysis was performed and the final multivariable model was selected after backward elimination method with the likelihood criteria. STATA version 12.0 was used for this analysis. In all statistical tests the level of significance was set at 0.05 and two-sided. The statistical analysis applied to the statistical package SPSS 17.0.

## Results

The initial sample size of the study consisted of 19,572 students. We gathered 19,027 questionnaires (participation rate: 97.2%). Non-participation was due to absence from school on the day of the study (e.g. illness, participation in sports or cultural activities). None of the students who were present at school refused to complete the questionnaire. One hundred and twenty three questionnaires were not suitable for analysis (e.g. age > 18 years old, silly/illogical/inadequately (<3 questions) answered: 1 million cig./day, tick both YES and NO answers). Finally the data of 18,904 questionnaires were encoded and statistically analysed.

We calculated the reproducibility rate of both questionnaires among 1,791 students (10% of the total number of the study’s schools). Reproducibility as assessed by the Cohen’s kappa statistic for the questionnaire of smokers and non-smokers was 0.87 (95% CI: 0.85, 0.90) and 0.88 (95% CI: 0.86, 0.91) respectively.

Concerning the whole study sample (n = 18,904, mean age ± SD: 15.3 ± 1.7 years), 2,685 students (14.2%) completed the smokers’ questionnaire which was equivalent with regular cigarette smoking. Of these, 2,245 (83.6%) reported everyday consumption (15.7 ± 10.6 cigarettes/day), 414 (15.4%) reported regular use but not every day (40.1 ± 72.9 cigarettes for the last month) and 26 students were current smokers but they had not given any information about their smoking habits. On the other hand 4,984 students of the current non-smokers reported that they had tried sometimes in the past (mean age ± SD: 13.9 ± 2.3 years). The percentage of lifetime cigarette use was 40.6% (7,669/18,904).

We initially compared smoking habits according to gender, school type and area of residence. Boys smoke more frequently than girls (16.4% vs 11.8%, p < 0.001, Table 1) while the percentage of students in high schools with technical education who reported regular smoking was more than double compared to that in general high schools (36.7% vs 17.8%, p < 0.001, Table 1). The above comparison did not include junior high schools due to low number of students in junior high schools with technical education (n = 198).

**Table 1:** Smoking habits according to gender, age group, area of residence and type of school

		**p-value (*χ***^**2**^**;df)**
Current smokers	2,685 (14.2%)	
Lifetime cigarette use	7,669/18,904 (40.6%)	
Males	1,610/9,806 (16.4%)	<0.001 (84.07;1)
Females	1,063/9,043 (11.8%)	
**12-15 years old** (junior high)	495/9,774 (5.1%)	
Males	293/4,944 (5.9%)	<0.001 (15.81;1)
Females	200/4,807 (4.2%)	
**16-18 years old** (high school)	2,158/8,960 (24.1%)	
Males	1,299/4,777 (27.2%)	<0.001 (55.50;1)
Females	851/4,163 (20.4%)	
**Urban area** (Thessaloniki)	873/5,465 (16%)	<0.001 (107.60;2)
Males	437/2,627 (16.6%)	0.207 (1.59;1)
Females	435/2,828 (15.4%)	
**Semirural areas** (counties’ capitals)	1,095/6,715 (16.3%)	
Males	725/3,755 (19.3%)	<0.001 (58.17;1)
Females	363/2,934 (12.4%)	
**Rural areas**	717/6,724 (10.7%)	
Males	448/3,424 (13.1%)*	<0.001 (44.21;1)
Females	265/3,281 (8.1%)	
General education (high schools)	1,100/6,167 (17.8%)	<0.001 (726.14;1)
Technical education (high schools)	1,058/2,793 (37.9%)	

In urban (city of Thessaloniki) and semirural areas (counties’ capitals) the incidence of smoking was higher than rural regions (p < 0.001, Table 1). It is worth noting that in urban area the percentage of smoking was similar between boys and girls (16.6% vs 15.4%, p = 0.207) while smoking prevalence among boys was significantly higher compared to girls in rural and semirural regions (p < 0.001 for both comparisons). The daily cigarette consumption was different among areas of residence (14.7 ± 9.8 for Thessaloniki, 16.5 ± 10.9 for semirural and 15.7 ± 11.1 for rural areas, p = 0.005, Kruskal-Wallis test).

Boys start smoking regularly at an earlier age compared to girls although the mean difference is only a few months (14.4 ± 1.9 vs 14.8 ± 1.5 years old, p < 0.001, Student’s *t*-test: 5.05, df: 2608). On the other hand the age of starting smoking did not differ among the three areas of residence (14.7 ± 1.7 vs 14.5 ± 1.8 vs 14.5 ± 1.8 years old respectively, p = 0.133, ANOVAF-test: 2.02, dfb/dfw: 2/2619).

We defined addiction to nicotine as consumption of more than 15 cigarettes/day and the first one within 30–60 minutes after awaking. We calculated that almost a third (32.8%) of the smokers fulfil the above criteria (Table 2). Curiosity and a way to deal with unpleasant feelings (e.g. nervousness, anxiety and anger) were the most reported causes for starting smoking (Table 3). Regarding the question about what smokers dislike in relation with smoking, the most popular answers were: the high price of cigarettes (55.7%), bad breath and clothes smell (50.3%) and harmful effect on my health (48.7%).

**Table 2:** Characteristics of current smokers about cigarette consumption

		**P-value (test; value; df)**
Age of starting regular smoking	14.6 ±1.7 years	
Everyday cigarette use (cigarettes/day)	2,245 (15.7 ±10.6)	
12–15 years old	334 (12.4 ±11.1)	<0.001 (Mann Whitney *U* test;8.17;na)
16–18 years old	1,885 (16.3 ±10.5)	
Males	1,365 (16.6 ±11.3)	<0.001 (Mann Whitney *U* test; 4.57;na)
Females	869 (14.3 ±9.4)	
Current smokers but not everyday use (cigarettes/last month)	414 (40.1 ±72.9)	
≥ 15 cigarettes per day	1,179/2,219 (53.1%)	
12–15 years old	128 (38.3%)	<0.001 (*χ*^2^;34.63;1)
16–18 years old	1,051 (55.8%)	
≥ 15 cigarettes per day and the first cigarette within 30–60 min after awaking in the morning	882 (32.8%)	
Tried to quit during the last 12 months	1,116/2,653 (42.1%)	
Their parents are aware that they smoke	1,706/2,647 (64.5%)	

na: not applicable.

**Table 3:** Reported causative factors that influenced them to become smokers or non-smokers

**Why did you start smoking?**		**Why do you not smoke?**	
Due to curiosity	56.6%	Smoking harms my health	81%
To deal with unpleasant feelings	38.7%	I hate cigarette’s smell	46.2%
Because my friends smoke	28%	It will affect my performance on sports	32.3%
To enjoy its taste	25.3%	I am afraid about cigarette dependence	28.8%
As a reaction to prohibition	15.8%	Most of my friends do not smoke	5.5%
I believe that smokers are fascinating personalities	8.5%	I can not afford it	2.6%

df: degrees of freedom, SD: standard deviation, BMI: Body mass index, OR: odds ratio, CI: confidence interval.

^a^ After backward elimination method with the likelihood ratio criteria.

Multivariable model: Wald *χ*^2^ = 2562.15;df = 15.

The associations of different factors with smoking status are presented in Table 4. Friends who smoke was proved to be the most important risk factor (OR: 10.01, 95% CI: 8.53-11.74, p < 0.001, Wald *χ*^2^ = 2562.15; df = 15). In multivariable analysis, the odds of smoking was higher in male gender, with higher age, with greater BMI, with frequent visits to internet cafes, when parents, siblings, friends and favorite artist smoked (Table 4). On the other hand, participation in sports, watching television and influence by health warning messages on cigarette packets act as protective factors against smoking (Table 4). An interesting finding was that knowledge about smoking related diseases was quite similar between the two groups. Higher percentages of non-smokers compared to smokers reported that anti-smoking measures are necessary (96% vs 68.3%, p < 0.001; Table 4). More specifically, the most popular answers were: prohibition of smoking in public places and workplaces (smokers: 31.5% vs non-smokers: 73.7%, p < 0.001, *x*^2^-test: 1580.31; df:1), prohibiting of cigarettes’ selling to people ≤17 years old (smokers: 43% vs non-smokers: 69.7%, p < 0.001, *x*^2^-test: 606.35; df:1) and informative campaign about the consequences of smoking on human health (smokers: 36.8% vs non-smokers: 58.4%, p < 0.001, *x*^2^-test: 361.71; df:1).

**Table 4:** Associations of anthropometric data, leisure activities, smoking in their social environment, educational level of their parents, knowledge about smoking related diseases and measures against smoking with smoking status

	**Smoking**		**Univariate analysis**			**Multivariable analysis**^**a**^		
	**yes**	**no**	**OR**	**95% CI**	**p-value (Wald*χ***^**2**^**;df)**	**OR**	**95% CI**	**p-value**
Male gender, n(%)	1610 (60.2)	8196 (50.7)	1.48	1.36, 1.61	<0.001 (81.9;1)	**1.31**	1.17,1.48	<0.001
Mean age in years (SD)	16.54 (1.25)	15.09 (1.63)	1.88	1.82, 1.95	<0.001 (1386;1)	**1.49**	1.42, 1.55	<0.001
Mean BMI in kg/m^2^ (SD)	21.88 (4.91)	20.62 (4.53)	1.06	1.05, 1.08	<0.001 (139.0;1)			
Sports activities (≥ 3 times/week), n (%)	779 (30.7)	7933 (50.6)	0.43	0.40, 0.47	<0.001 (334.4;1)	**0.59**	0.53, 0.67	<0.001
Internet cafes (≥ 3 times/week), n (%)	821 (32.3)	2556 (16.3)	2.48	2.25, 2.73	<0.001 (350.1;1)	**1.53**	1.35, 1.74	<0.001
Watch TV everyday, n (%)	927 (36.5)	6068 (38.7)	0.89	0.82, 0.98	0.013 (6.23;1)	**0.74**	0.66, 0.84	<0.001
Smokers in the social environment, n (%)								
Father	1783 (66.6)	8383 (51.7)	1.83	1.68, 2.00	<0.001 (186.1;1)	**1.37**	1.22, 1.54	<0.001
Mother	1377 (51.5)	6056 (37.4)	1.77	1.63, 1.93	<0.001 (180.7;1)	**1.26**	1.13, 1.41	<0.001
Siblings	1108 (41.4)	2363 (14.6)	4.02	3.68, 4.40	<0.001 (932.2;1)	**2.24**	1.99, 2.51	<0.001
Friends	2443 (91.2)	5229 (32.3)	20.96	18.26, 24.06	<0.001 (1864;1)	**10.01**	8.53, 11.74	<0.001
Favorite teacher	793 (29.6)	3956 (24.4)	1.31	1.20, 1.44	<0.001 (33.5; 1)			
Favorite artist	914 (34.3)	3750 (23.1)	1.72	1.58, 1.88	<0.001 (143.8;1)	**1.18**	1.04, 1.33	0.009
Educational level of the parents, n (%)								
Father: University	785 (29.7)	6150 (38.7)	1.00	reference	(78.2;2)			
Father: High School	1422 (53.8)	7816 (49.2)	**1.38**	1.26, 1.52	<0.001			
Father: Elementary school	435 (16.5)	1923 (12.1)	**1.74**	1.52, 1.98	<0.001			
Mother: University	865 (32.9)	6546 (41.3)	1.00	reference	(90.1;2)			
Mother: High School	1392 (52.9)	7857 (49.6)	**1.29**	1.17, 1.41	<0.001			
Mother: Elementary school	375 (14.2)	1446 (9.1)	**1.93**	1.68, 2.21	<0.001			
Smoking is related with, n (%)								
Lung cancer	2244 (90.2)	15140 (94.0)	.57	0.49, 0.66	<0.001 (53.6;1)	**0.74**	0.61, 0.91	0.003
Chronic bronchitis	085 (43.7)	7553 (46.9)	0.86	0.79, 0.94	0.001 (11.4;1)	**0.86**	0.77, 0.97	0.012
Heart diseases	1485 (59.7)	10682 (66.3)	0.74	0.68, 0.81	<0.001 (45.9;1)	**0.83**	0.74, 0.93	0.002
Stroke	840 (33.8)	5689 (35.3)	0.91	0.83, 0.99	0.036 (4.4;1)			
Messages in the cigarette affect them, n (%)	516 (20.3)	9159 (58.1)	0.19	0.17, 0.21	<0.001 (1022;1)	**0.42**	0.37, 0.48	<0.001
Anti-smoking measures are necessary, n (%)	1734 (68.3)	15035 (96.0)	0.09	0.08, 0.10	<0.001 (1615;1)	**0.21**	0.18, 0.24	<0.001

Finally we tried to correlate intensity of smoking (number of cigarettes per day) with various social factors reported in the questionnaires. Smoking status of father (14.7 ±10.2 cig./day if father does not smoke vs 16.2 ±10.8 cig./day if father smokes, p = 0.001; Mann–Whitney *U* test: -3.38), mother (14.8 ±10 vs 16.6 ±11.1, p < 0.001), siblings (14.7 ±10.2 vs 17.1 ±11.1, p < 0.001; Mann–Whitney *U* test: -3.97), educational level of father (15.4 ±11.3 cig./day for the university degree vs 17.7 ±12.4 cig./day for the lowest score elementary school, p = 0.001; Mann–Whitney *U* test: -3.37) and educational level of mother (15.1 ±10.5 vs 17.6 ±11.5, p = 0.001; Mann–Whitney *U* test: -3.39) were associated with smoking intensity of the adolescents. We also found that those who visit more frequently internet café (17.8 ±12.2 cig./day vs 13.7 ±8.7, p < 0.001; Mann–Whitney *U* test: -6.10) and were not influenced by the messages on cigarette packets (16.2 ±10.5 cig./day vs 13.6 ±10.6, p < 0.001; Mann–Whitney *U* test: -5.80) used to smoke more. Adolescents whose parents know that they smoke used to consume more cigarettes daily (17.8 ±10.9 vs 10.9 ±8.1, p < 0.001; Mann–Whitney *U* test: -15.98).

## Discussion

To our current knowledge this is the largest – according to the number of participants – epidemiological study in Greece about cigarette smoking among adolescents. The main findings of the present study are: a) 14.2% of the adolescents (mean age ± SD: 15.3 ± 1.7 years) reported regular smoking (24.1% in the age group 16–18 years), b) 40.6% reported lifetime cigarette use, c) 84.2% of the current smokers reported daily cigarette use, d) students who live in urban and semirural areas smoke more frequently than those in rural areas, e) students in technically oriented schools smoke twice as frequent compared to those in general education, f) we identified as risk factors for smoking: male gender, age, friends who smoke, frequent visits to internet cafés, parents, siblings and favourite artist who smoke, educational level of the parents (Table 4), g) we identified as protective factors against smoking: participation in sports, watching television and influence by health warning messages on cigarette packets (Table 4). The same factors are related with the intensity of smoking (cigarettes/day) as well as the presence of parents who are aware that their child smokes.

In a cross-sectional national survey in the USA among high school students it was found that the percentage of current smokers was 20% (21.3% for males and 18.7% for females) in 2007 while 8.1% reported smoking on 20 or more days during the last 30 days [8]. In our study the percentage of current smokers among high school students was 24.1% (27.2% for males and 20.4% for females) while the percentage of everyday smokers was 21% (1,885/8,960). In the Global Youth Tobacco Survey [9], which was conducted among students 13–15 years old, it was found that 9.5% of the participants were current smokers (19.2% for eastern European countries, 10.4% cigarette smoking for Greece in 2005 and 4.9% for the eastern Mediterranean region). In our study the percentage of current smokers among junior high school students was 5.1%.

Several studies have concluded that the risk for an adolescent to become a current smoker is increased if their parents [10,27-29], siblings [28,29] or best friends [28,29] smoke. In a recent meta-analyses which included 58 studies [30], the relative odds of uptake of smoking in children were increased significantly if at least one parent smoked (OR: 1.72, 95% CI 1.59-1.86), more so by smoking by the mother (OR 2.19, 95% CI 1.73-2.79) than the father (OR: 1.66, 95% CI 1.42-1.94) while smoking by a sibling increased the odds of smoking uptake by 2.30 (95% CI 1.85-2.86). In a recent review that investigated the relationship between participation in sports and smoking among adolescents, 14 out of 15 studies had found an inverse relationship [25]. The above findings are in accordance with our conclusions.

A recent study among adolescents in high schools in Thessaloniki, Greece found that physical activity was negatively correlated with smoking, whereas drinking alcohol and low parental education were positively correlated [31]. Damianaki et al [32] conducted a cross-sectional study in a sample of 924 students (12–18 years old) in a semi-urban area in Crete, Greece and found that 11.4% of the total sample was daily smokers (2,245/18,904 = 11.9% in our study) while there was positive relationship between current smoking and having brother or sister smoking (odds ratio: 2.7 (95% CI: 1.7-4.4) and 1.8 (1.1-3.3) respectively), having more than three friends who were smokers (OR: 2.6 (95% CI: 2–3.4) and last school grade [OR:1.4 (95% CI: 1.2-1.7)]. The Global Youth Tobacco Survey (GYTS) was conducted in Greece [33,34] among 6,378 students (13–15 years old) during the academic year 2004–5. The authors found that 16.4% of the students reported being current users of tobacco products, 10.4% were current smokers of cigarettes while male gender (OR: 1.62; 95% CI: 1.08-3.08), parental smoking (OR: 2.59; 95% CI: 1.45-5.89), and having pocket money ≥ 16 Euros (OR:2.64; 95% CI: 1.19-5.98) proved to be independent risk factors associated with current cigarette smoking. In our study, which included adolescents 12–18 years old, the prevalence of current cigarette smoking was 14.2% while we also found similar risk factors as the above mentioned studies. Additionally we identified some important protective factors.

Regarding risk factors a study of Russian adolescents found that behavioral activation increased conflict with parents and a tendency to go to clubs and bars, both of which increased cigarette use [35]. In the present study frequent (≥3 times/week) visits to internet cafés significantly increased the risk for smoking. Low socioeconomic status has been associated with greater cigarette smoking in several studies [36]. In our study educational level of the parents, which is usually an indirect index of socioeconomic status, was not related with adolescents’ cigarette use.

A recent systematic review [37] showed that media exposure was associated with increased risk of smoking initiation even though we found that watching television everyday was a protective factor against smoking. We hypothesized that prohibition of cigarette advertising on TV as well as educational programs about smoking related diseases could be an explanation. A report by Centers for Disease Control and Prevention [38] in 14 countries about the effectiveness of cigarette package health warnings concluded that percentage of adult smokers thinking about quitting because of the warnings was >50% in six countries. Similarly we found that messages on cigarette packets act as a protective factor against smoking for adolescents.

We should mention that using two different questionnaires is not a common practice for most epidemiological studies and this could be considered a limitation. We decided to use two questionnaires because we thought that this would increase response rate and minimize time required for completion. As the percentages of current and lifetime cigarette smokers among Greek adolescents were quite high effective smoking prevention programs are urgently needed. The present study proved that many risk and protective social factors should be taken into account.

## Competing interest

The authors declare that they have no competing interests.

## Authors’ contribution

DGS, DTP, DC, AHB, and LTS conception and design, acquisition of data or analysis and interpretation of data. EK, and KG, draft the article or revise it critically for important intellectual content. SK and CK final approval of the version published. All authors read and approved the final manuscript.

## Supplementary Material

Additional file 1 Appendix (questionnaires for smokers and non-smokers). Click here for file
