# Supplementary material for: Smoking among adolescents in Northern Greece: a large cross-sectional study about risk and preventive factors
Source: Subst Abuse Treat Prev Policy. 2012 Sep 10;7:38. doi: 10.1186/1747-597X-7-38 (PMC3511804; doi:10.1186/1747-597X-7-38)
Supplement: Additional file 1 — Appendix (questionnaires for smokers and non-smokers). [file 1747-597X-7-38-S1.doc]

**Appendix (questionnaires)**

**NON-Smoker**

**Gender: Boy Girl**

**Age Height (cm) Weight (kg)**

**How old were you when you tried your first cigarette?**

**How old were you when you started smoking?**

**In case you smoke everyday, how many cigarettes do you smoke per day?**

**In case you don’t smoke everyday, how many cigarettes have you smoked the last month?**

**Do you smoke the first cigarette in the morning within 30-60 min after awakening?**

YES 🞏 NO 🞏

**Have you tried to quit smoking during the last 12 months?**

YES 🞏 NO 🞏

**Do your parents know that you smoke?**

YES 🞏 NO 🞏

**Parent’s education:**

Father Mother

Elementary school 🞏 🞏

Junior high-High school 🞏 🞏

College-University 🞏 🞏

**Who of the following persons smoke?**

🞏 Your father 🞏 Your friends

🞏 Your mother 🞏 Your favorite teacher

🞏 Your brother/sister 🞏 Your favorite artist

**Why did you start smoking?**

🞏 To enjoy its taste

🞏 To deal with unpleasant feelings

🞏 To keep my weight low

🞏 Because my friends smoke

🞏 As a reaction to prohibition

🞏 I believe that smokers are fascinating personalities

🞏 Because I have grown up

🞏 Due to curiosity

**Are you affected by the messages on the packets of cigarettes?**

YES 🞏 NO 🞏

**Which of the following diseases do you believe are related with smoking?**

🞏 Lung cancer 🞏 Heart diseases

🞏 Chronic bronchitis 🞏 Stroke

**Do you take part in sports activities?**

At least 3 times/week 🞏 Sometimes 🞏 Never 🞏

**Do you watch television?**

Everyday 🞏 Sometimes 🞏 Never 🞏

**Do you visit internet cafés?**

At least 3 times/week 🞏 Sometimes 🞏 Never 🞏

**Which of the following do you dislike regarding smoking?**

🞏 Cigarettes are expensive

🞏 Smoking harms my health

🞏 My clothes and my breath smell bad

🞏 I am not accepted by a company of non-smokers

🞏 I don’t like cigarette dependence

🞏 It affects my performance on sports

**Do you believe that anti-smoking measures are necessary?**

YES 🞏 NO 🞏

**If YES**

**What do you believe should be done for the problem of cigarette smoking?**

🞏 Informative campaign about the consequences of smoking on health

🞏 Prohibition to advertisement

🞏 Increase in cigarette’s price

🞏 Prohibition of selling cigarettes to adolescents (under 17 year-old)

🞏 Prohibition of smoking in public buildings and workplace

🞏 Prohibition of smoking for famous persons when they are in the public eye

**non-Smoker**

**Gender: Boy Girl**

**Age Height (cm) Weight (kg)**

**What the reasons that you don’t smoke?**

🞏 I am sure that smoking harms my health

🞏 I can not afford it

🞏 Because most of my friends do not smoke

🞏 I hate cigarette’s smell

🞏 I am afraid about cigarette dependence

🞏 It will affect my performance on sports

**Have you ever tried?**

YES 🞏 NO 🞏

**If YES in what age?**

**Parent’s education:**

Father Mother

Elementary school 🞏 🞏

Junior high-High school 🞏 🞏

College-University 🞏 🞏

**Who of the following persons smoke?**

🞏 Your father 🞏 Your friends

🞏 Your mother 🞏 Your favorite teacher

🞏 Your brother/sister 🞏 Your favorite artist

**Are you affected by the messages on the packets of cigarettes?**

YES 🞏 NO 🞏

**Which of the following diseases do you believe are related with smoking?**

🞏 Lung cancer 🞏 Heart diseases

🞏 Chronic bronchitis 🞏 Stroke

**Do you take part in sports activities?**

At least 3 times/week 🞏 Sometimes 🞏 Never 🞏

**Do you watch television?**

Everyday 🞏 Sometimes 🞏 Never 🞏

**Do you visit internet cafés?**

At least 3 times/week 🞏 Sometimes 🞏 Never 🞏

**Do you believe that anti-smoking measures are necessary?**

YES 🞏 NO 🞏

**If YES**

**What do you believe should be done for the problem of cigarette smoking?**

🞏 Informative campaign about the consequences of smoking on health

🞏 Prohibition to advertisement

🞏 Increase in cigarette’s price

🞏 Prohibition of selling cigarettes to adolescents (under 17 year-old)

🞏 Prohibition of smoking in public buildings and workplace

🞏 Prohibition of smoking for famous persons when they are in the public eye
